# Effectiveness of Buffered Lidocaine With Sodium Bicarbonate Versus Conventional Lidocaine as Local Anesthetic Solutions for Pain Management in Pediatric Dentistry: A Randomized, Split‐Mouth, Crossover, Double‐Blind Clinical Trial

**DOI:** 10.1002/cre2.70435

**Published:** 2026-08-25

**Authors:** Abolfazl Shiri Varnamkhasti, Sajjad Amiri, Raziyeh Shojaeipour, Milad Mollaali

**Affiliations:** ^1^ Oral and Dental Diseases Research Center, Department of Pediatric Dentistry, School of Dentistry Kerman University of Medical Sciences Kerman Iran; ^2^ Department of Pediatric Dentistry Isfahan University of Medical Sciences Isfahan Iran; ^3^ Department of Biology, Faculty of Science University of Sistan and Baluchestan Zahedan Iran

**Keywords:** lidocaine, local anesthesia, pain management, sodium bicarbonate

## Abstract

**Objectives:**

Managing injection pain is critical for pediatric patient cooperation. The clinical benefit of using 7.5% sodium bicarbonate‐buffered lidocaine, compared to its unbuffered counterpart, has not been extensively studied in children using a controlled split‐mouth design. Hence, this clinical trial aimed to compare the pain control efficacy of buffered versus conventional lidocaine in pediatric dentistry, assessing pain during injection, anesthetic depth, and anesthetic onset.

**Material and Methods:**

A randomized, split‐mouth, crossover, double‐blind clinical trial was conducted involving 53 healthy children referred to the Kerman Dental School. Children requiring local anesthesia for the same dental procedure on two molars of the same name in one jaw were randomly assigned to Group 1 (first session: conventional 2% lidocaine; second session: buffered 2% lidocaine with 7.5% sodium bicarbonate) and Group 2 (reverse sequence). The two sessions were separated by a 1‑week washout period. Pain during injection was assessed using the SEM (Sound, Eye, Motor) scale and the FPS‐R (Facial Pain Scale‐Revised), and pulse rate changes were monitored. The SEM scale also assessed both the anesthetic onset and depth.

**Results:**

Buffered lidocaine resulted in significantly lower SEM scores during injection (a 17% reduction; Exp(*β*) = 1.17, 95% CI [1.10, 1.23], *p* < 0.001) and faster anesthetic onset (a 6% improvement; Exp(*β*) = 1.06, 95% CI [1.01, 1.11], *p* = 0.011) compared with conventional lidocaine. These improvements did not extend to subjective pain scores (FPS‑R), where no significant difference was observed. Pulse rate increased similarly after the injection of either local anesthetic formulation, with no treatment‑dependent hemodynamic effect.

**Conclusions:**

The administration of buffered lidocaine with 7.5% sodium bicarbonate significantly improved objective behavioral outcomes, as evidenced by lower SEM scores and a quicker onset, compared to conventional lidocaine. While these findings did not extend to subjective pain scores, the reduction in physical distress behaviors advocates for its clinical use in pediatric dental procedures.

**Trial Registration:**

IRCT20241119063775N1.

## 1 Introduction

Effective pain management is important in pediatric dentistry because inadequate pain control can lead to fear, anxiety, and a lack of cooperation, which not only affects the child's comfort during dental visits but also impacts their willingness to seek future dental care. Among the various procedures in pediatric dentistry, injection is often considered the most challenging, highlighting the need for effective pain management strategies. Experts agree that effective pain management is key to successfully managing a child's behavior. Inadequate pain management can cause patients to have negative reactions, avoid seeking treatment, and develop long‐term dental anxiety. On the other hand, properly managing pain helps build trust, encourages patients to follow treatment plans, and leads to more positive dental experiences (Remi et al. 2023; Alsarheed 2011; Kharouba et al. 2023; Kumar et al. 2019).

Local anesthetics play a crucial role in reducing pain during dental treatments, which in turn helps improve a child's cooperation and the overall success of the procedure (Monteiro et al. 2020). Research indicates that buffering lidocaine to make its pH closer to normal body levels can help reduce the discomfort experienced during injections (Cepeda et al. 2010). Lidocaine with epinephrine demonstrated an acidity level approximately one thousand times higher than that of subcutaneous tissue; furthermore, the inclusion of sodium bicarbonate offers a practical and economical approach for reducing injection pain (Meincken et al. 2019; Frank and Lalonde 2012).

Accurate pain assessment in pediatric dental patients is critical for delivering effective pain medication and achieving the best possible treatment outcomes. Various methods are developed to understand how children feel pain, including checking physiological signs such as monitoring pulse rate; using behavioral tools such as the Sound, Eyes, Motor (SEM) scale; and self‐report measures such as the use of Wong‐Baker FACES Pain Rating Scale (WBFPRS) as a Faces Pain Scale‐Revised (FPS‐R) assay. Self‐report measures are particularly popular in pediatric studies due to their reliability and ease of use (Hicks et al. 2001; Garra et al. 2010; Wright et al. 1991; Mittal et al. 2019).

Therefore, to address the challenges of pain management in pediatric patients, we aim, in this double‐blind, crossover, split‐mouth clinical trial, to compare conventional lidocaine and buffered lidocaine local anesthesia (LA) approaches, focusing on their effectiveness in reducing pain in Iranian pediatric dental patients. Specifically, we will assess the differences between the two methods in terms of pain severity during injection, LA depth, and duration of LA.

## 2 Methods

### 2.1 Study Design

This double‐blind randomized split‐mouth crossover clinical trial was conducted in the Department of Pediatric Dentistry at Kerman School of Dentistry, Kerman, Iran, from September 2023 to June 2024. Ethics approval was obtained from the Ethics Committee of Kerman University of Medical Sciences under the code: IR.KMU.REC.1401.161. The trial was registered retrospectively at the Iranian Registry of Clinical Trials (ID: IRCT20241119063775N1) on February 19, 2025. Registration occurred after the study and analysis were complete because the research team was not fully aware of the prospective registration requirement when the study began. We populated the registration entry directly from the protocol approved by the Kerman Research Ethics Committee on June 6, 2022, which pre‑specified all endpoints, the sample size, randomization, and the statistical analysis plan, and which is detailed in the manuscript and the CONSORT flowchart. No changes were made to these elements at any stage. The trial was designed and reported in accordance with the CONSORT 2010 statement (Schulz et al. 2010).

### 2.2 Eligibility Criteria

The inclusion criteria for children in the study were as follows: General health considerations included: (1) Physical health assessment ruling out acute or chronic illnesses, allergies, respiratory infections, and fever, with no antibiotics taken in the past 3 weeks. (2) Mental health evaluation ensuring the child resides with both parents and exhibits minimal stress and anxiety levels, as measured by the Beck Anxiety Inventory (BAI) Scale (Beck et al. 1988; Kaviani and Mousavi 2008), which comprises 21 self‐reported items rated on a four‐point scale. This scale is utilized to assess the severity of physical and cognitive anxiety symptoms over the past week, with scores ranging from 0 to 63, categorized as “minimal anxiety (0–7),” “mild anxiety (8–15)”, “moderate anxiety (16–25)”, and “severe anxiety (26–63)” (Dávid et al. 2023). (3) The child must demonstrate a Frankl Behaviour Rating Scale of 3 or 4 (Frankl et al. 1962). (4) The child should be in the primary or mixed dentition phase, as each child's condition is compared to their own baseline (split mouth methodology). (5) No previous history of receiving LA for dental procedures. (6) The child is not currently on medications that interfere with local anesthetic solutions or sodium bicarbonate, or medications that alter pain perception, such as analgesics or tranquilizers. (7) Having two molars with the same name in one jaw that require LA for similar treatment.

The criteria for excluding children from the study were: (1) The child is uncooperative during the injection or during dental treatment in the first or second treatment session. (2) The patient or his/her parents refuse to continue participating in the study. (3) The presence of any lesion in the injection area, such as a fistula, abscess, or wound. (4) Lack of obtaining LA and the need for a second injection. (5) Treating two teeth with the same name in the same jaw should not be similar.

### 2.3 Data Collection

All qualified participants attended interviews and clinical assessments to record demographic data. One pediatric dental resident performed all clinical examinations.

### 2.4 Clinical Intervention

*Preparation of the anesthetic solutions*: both the buffered and the conventional solutions were prepared from standard 1.8 mL cartridges of 2% lidocaine with epinephrine 1:80,000 (Darou Pakhsh Co., Tehran, Iran). Using a Hamilton gastight syringe (Hamilton Co., Nevada, USA), 0.18 mL of the solution was extracted from the cartridge and replaced with an equal volume of either 7.5% sodium bicarbonate (Samen Co., Mashhad, Iran) for the buffered formulation or sterile distilled water (Samen Co., Mashhad, Iran) for the conventional formulation. This resulted in a 1:10 dilution of the additive and a final total volume of 1.8 mL (Saatchi et al. 2015). Both cartridges were then agitated five times at room temperature to ensure complete homogenization and to confirm the absence of any precipitate. A qualified dental resident who was not involved in the clinical procedure prepared the two formulations and assigned them randomized codes, thereby maintaining blinding of the operator and outcome assessors. All LA injections were performed by a single operator (SA).

*Injection procedure*: an aspirating syringe equipped with a short (20 mm) 27‑gauge needle (Soha Co., Tehran, Iran) was used for all injections. The solution was injected at a controlled, slow rate of approximately 1 mL/min to minimize pressure‑induced pain and to ensure uniform distribution of the anesthetic. The same injection technique was used in both sessions.

*Washout period and sequence*: a 1‑week washout period was enforced between the two treatment sessions. Children were randomly assigned to one of two groups:

- Group 1: conventional 2% lidocaine (Session 1) followed by buffered 2% lidocaine with 7.5% sodium bicarbonate (Session 2).
- Group 2: buffered 2% lidocaine with 7.5% sodium bicarbonate (Session 1) followed by conventional 2% lidocaine (Session 2).

### 2.5 Outcomes

The primary outcome of this study was pain severity during the local anesthetic injection, assessed using the SEM scale, the FPS‑R, and changes in pulse rate. Secondary outcomes were the time to onset of LA and anesthetic depth, both documented using the SEM criteria once the dental procedure began.

### 2.6 Measurements

#### 2.6.1 Part I. The Child's Comfort During the Injection Was Assessed Using Three Methods

**Figure 1:**
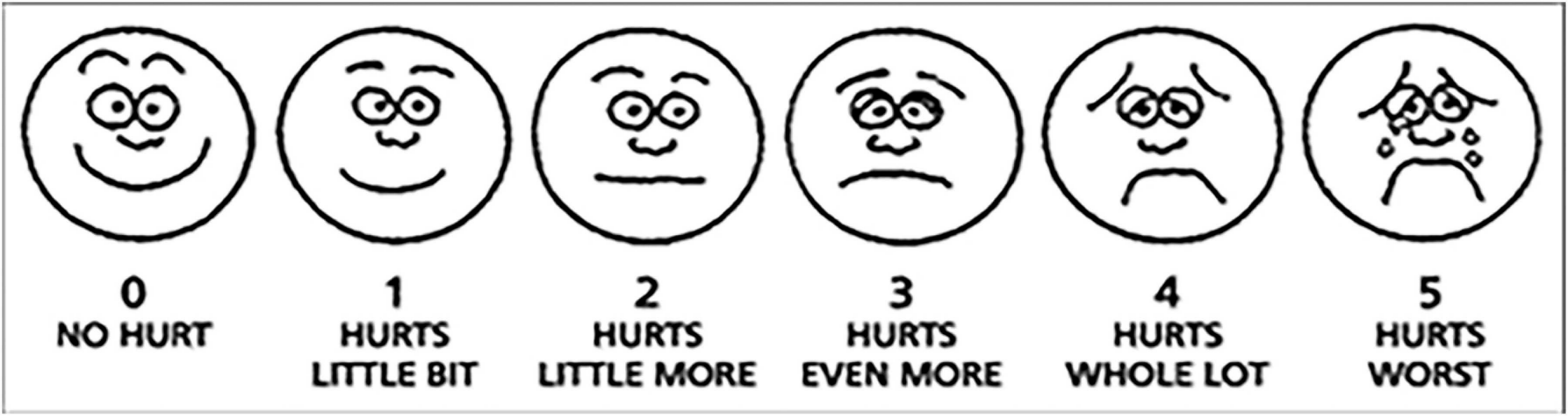
Wong‐Baker FACES scale. This method is used to evaluate the Faces Pain Scale‐Revised (FPS‐R) during the first and second sessions of local anesthesia injections.

1. *Heart (pulse) rate*: It is a physiological index; it was measured using a finger pulse oximeter (Model PO16; Brisk Medical Co., Germany) and recorded in beats per minute (bpm). Readings were taken both prior to and following the injection during two phases of treatment to assess levels of anxiety and pain.

2. *The SEM scale*: It is a behavioral observation. A trained technician recorded children's voice, eye, and body movements during LA injection. The detailed criteria for the SEM scale are described earlier. Briefly, no pain (score = 1), mild discomfort (score = 2), moderate pain (score = 3), and Severe pain (score = 4). These scores were measured for these three parameters (sound, eye, and motor).

3. *FPS‐R*: The WBFPRS was employed for the subjective assessment of pain as a powerful tool in the FPS‐R assay. The scale is introduced to the child as a self‐report index immediately after the LA injection, with each face corresponding to a specific numerical value. This scale ranged from 0 (no hurt) to 5 (hurts worst); the WBFPRS tool is provided in Figure 1 (Ghaderi et al. 2013).

#### 2.6.2 Part II. Assessment of the Initiation of LA

Five minutes after administering the injection, 0.5 mm of the catheter tip was carefully inserted into the mesial papilla of the target tooth. This process was repeated every minute up to five times until the child demonstrated either comfort or mild discomfort, as assessed using the SEM scale. The time at which this response occurs is documented as the onset of LA.

#### 2.6.3 Part III. Evaluation of Anesthetic Depth

Once LA was achieved, the dental procedure began on the targeted tooth, and the child's response throughout treatment was documented according to the SEM criteria.

### 2.7 Sample Size

The minimum number of participants required for this investigation was estimated using G*Power software 3.1.9.7 (Heinrich‐Heine‐Universität Düsseldorf, Düsseldorf, Germany), based on prior research (Sravya and Nirmala 2025), with a power of 0.8, an alpha level of 0.05, and an effect size of 0.8. The estimated total population was 52, with each group comprising 26 participants.

### 2.8 Randomization, Sequence Generation, and Allocation

Participants were allocated to the two treatment groups utilizing a randomized block approach. To mitigate the influence of potential confounding variables, participants were initially sorted by age and gender to create homogeneous blocks of four. A computer‑generated random allocation sequence, prepared by one of the investigators (R.S.), was used within each block to allocate two participants to Group 1 and two to Group 2. Once blocks are created, treatments are randomly assigned within each block to ensure each treatment appears only once per block. This technique guaranteed an equitable distribution of essential traits across the two groups during the trial.

### 2.9 Blinding

To ensure double‑blinding, a qualified dental resident not involved in the clinical procedure prepared the two formulas and assigned them randomized codes. The operator performing the injection and the technician recording the SEM scores were blinded to the solution type. While complete blinding of pediatric participants is challenging, identical syringes and standardized administration were used to minimize identification.

### 2.10 Statistical Analysis

Data normality was evaluated using the Shapiro–Wilk test. Continuous variables are reported as mean (SD) or as the median (IQR), depending on their distribution. Depending on normality, appropriate parametric or non‐parametric tests were chosen for group comparisons. Paired Samples *t*‐test or Wilcoxon Signed‐Rank test was used for within‐group comparisons. In addition, the Independent Samples *t*‐test or the Mann–Whitney *U* test was used to compare two independent groups. One‐way ANOVA test or Kruskal–Wallis test was applied for more than two groups. Categorical variables were analyzed with the Chi‐Square test.

Generalized Linear Mixed Models (GLMMs) accounted for the repeated‑measures crossover design via a subject‑specific random intercept. SEM during injection, SEM depth, and onset time were fitted with Gamma (log link) distributions; pulse rate change with a normal (identity link) distribution; and the ordinal FPS‑R with a multinomial cumulative logit model. Fixed effects included treatment, group, session, gender, age, tooth type, jaw, and dental procedure.

Model fit was evaluated with the corrected Akaike Information Criterion (AICc), Bayesian Information Criterion (BIC), the Gamma scale parameter (φ), pseudo‐*R* ^*2*^ values, and graphical residual diagnostics.

Analyses were performed in SPSS 27 (IBM Corp., Armonk, NY). Graphs were produced with GraphPad Prism 10.2.3 (GraphPad Software, San Diego, CA, USA). A two‑sided *p* < 0.05 was considered significant.

## 3 Results

### 3.1 Demographic Information of the Trial

**Figure 2:**
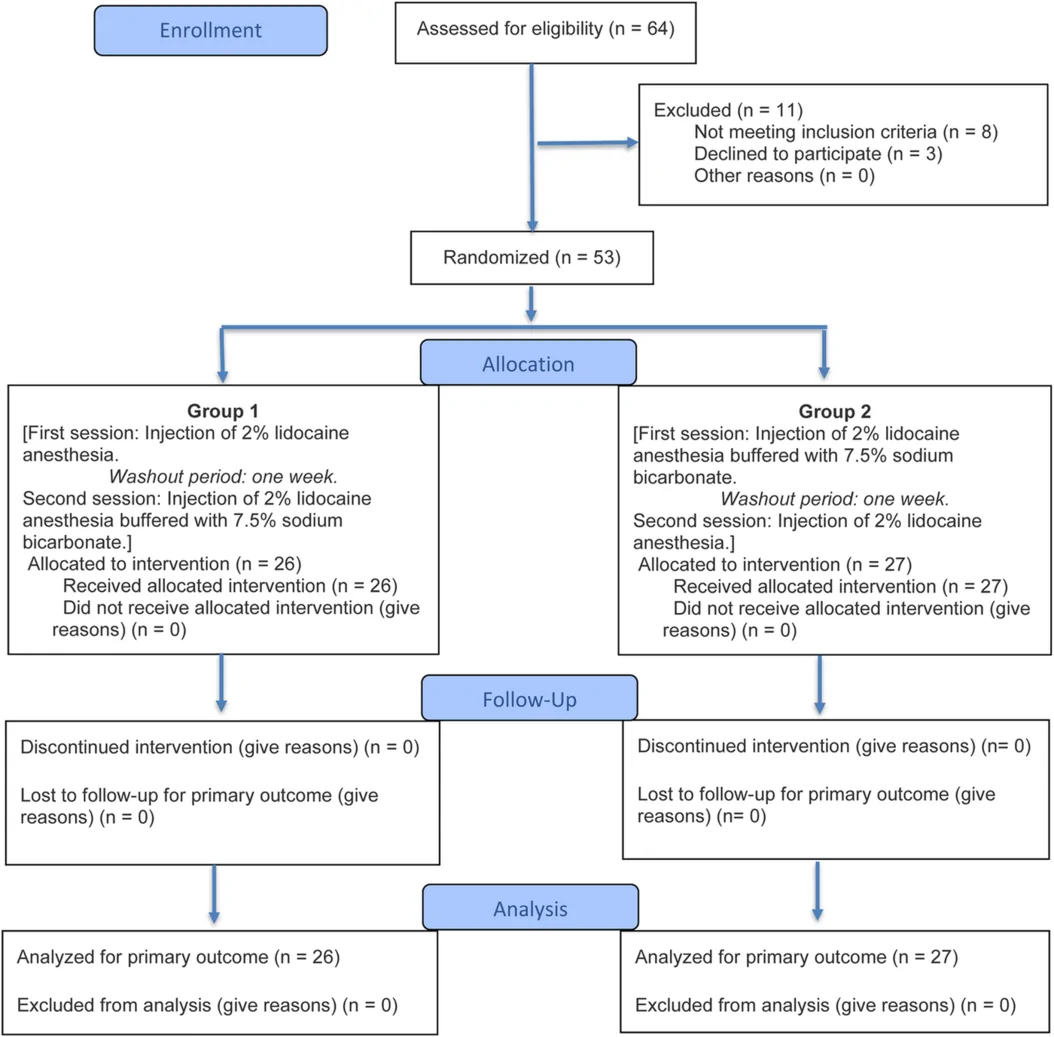
CONSORT flowchart.

The CONSORT flow diagram is provided in Figure 2.

Comprehensive information about the participants is listed in Table 1.

**Table 1:** Demographic information of participants.

Variables	Group 1 (*N* = 26)	Group 2 (*N* = 27)	*χ* ^2^	*p* a	Cramer's *V* b
Gender			0.182	0.669	0.059
Female	11 (42.3%)	13 (48.1%)			
Male	15 (57.7%)	14 (51.9%)			
Tooth target			0.508	0.776	0.098
D	12 (46.2%)	10 (37.1%)			
E	6 (23.1%)	8 (29.6%)			
6	8 (30.7%)	9 (33.3%)			
Jaw			0.506	0.477	0.098
Maxilla	16 (61.5%)	14 (51.9%)			
Mandible	10 (38.5%)	13 (48.1%)			
Dental procedures			2.274	0.81	0.207
Extraction	5 (19.2%)	8 (29.6%)			
Crown	1 (3.8%)	0 (0%)			
Pulpotomy	8 (30.8%)	7 (26%)			
Pulpectomy	3 (11.6%)	2 (7.4%)			
Apexogenesis	1 (3.8%)	2 (7.4%)			
Restoration	8 (30.8%)	8 (29.6%)			
Age (mean year (SD))	7.46 (2.27)	7.3 (2.41)	0.257^c^	0.799^d^	0.071^b^, ^e^

^a^ Chi‐square test of independence.

^b^ Effect size.

^c^ *t* statistics.

^d^ Independent samples *t*‐test.

^e^ Cohen's *d*.

### 3.2 Evaluation of the Normality of Data

Normality was evaluated using the Shapiro–Wilk test. The results demonstrated that all continuous variables, except for age and change in pulse rate, exhibited significant departures from normality (*p* < 0.05). Accordingly, nonparametric tests were employed for variables that failed to meet the assumptions of normality. In contrast, parametric tests were retained for age and pulse rate change due to their distributional conformity.

### 3.3 Pain During the Injection of LA

**Figure 3:**
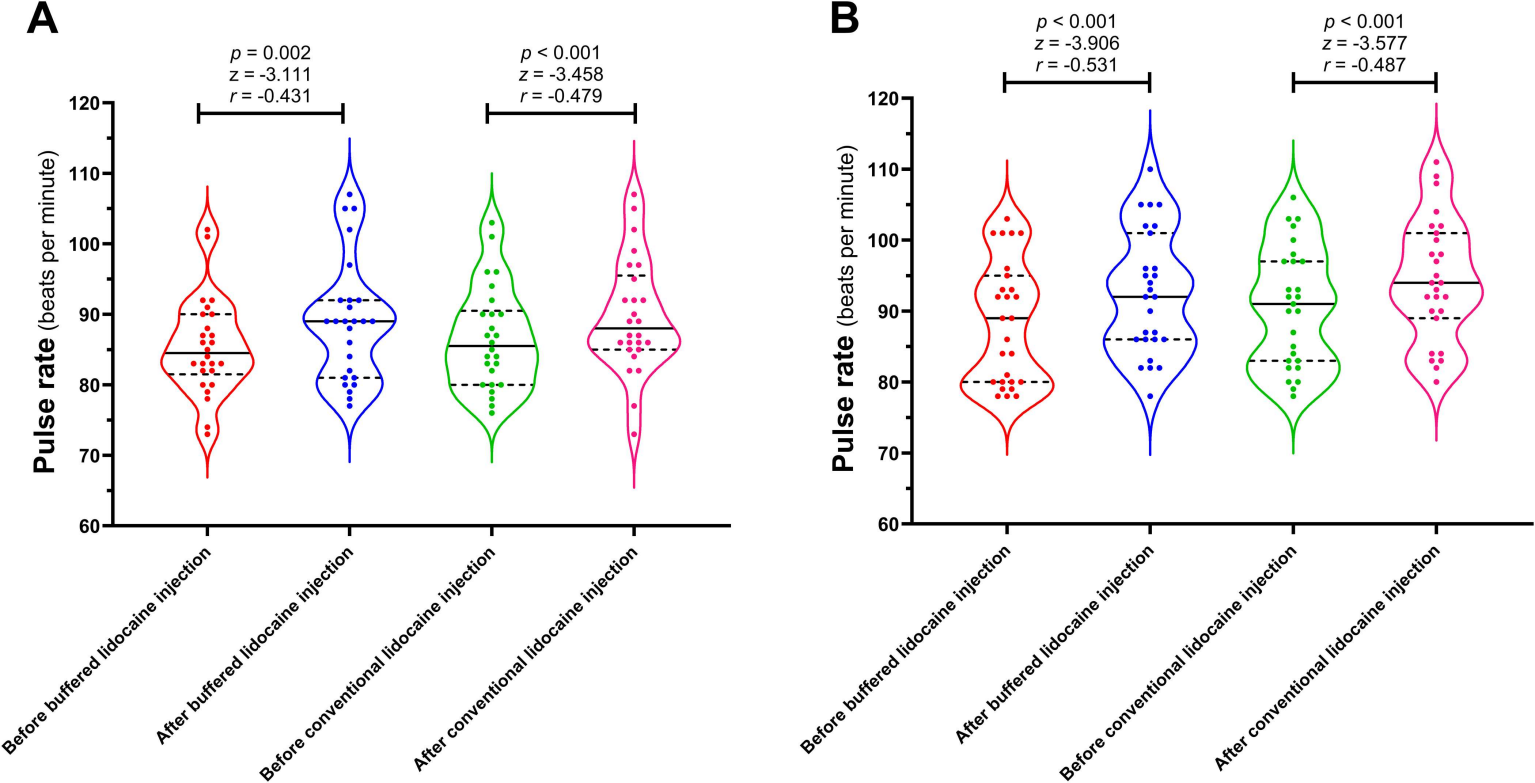
Violin charts of pulse rate (beats per minute) of participants before and after local anesthesia injections at the first and second sessions. Panel A shows Group 1, and Panel B shows Group 2. Each individual is represented as a colored circle. Straight and dashed lines indicate medians and quartiles, respectively. Data were analyzed with the Wilcoxon signed ranks test; *r* values indicate effect size.

Pulse rates were recorded for each group before and after LA injection in each session. The comparison of pulse rate is provided in Figure 3.

Pulse rate increased significantly from before to after LA injection across all conditions. For Group 1 (Figure 3A), pulse rate rose from a median of 85.5 bpm (IQR 80.0–90.5) to 88.0 bpm (85.0–95.5) after conventional lidocaine (Wilcoxon *Z* = −3.458, *p* < 0.001, *r* = −0.479) and from 84.5 bpm (81.5–90.0) to 89.0 bpm (81.0–92.0) after buffered lidocaine (*Z* = −3.111, *p* = 0.002, *r* = −0.433). For Group 2 (Figure 3B), pulse rate increased from 89.0 bpm (80.0–95.0) to 92.0 bpm (86.0–101.0) after buffered lidocaine (*Z* = −3.906, *p* < 0.001, *r* = −0.531) and from 91.0 bpm (83.0–97.0) to 94.0 bpm (89.0–101.0) after conventional lidocaine (*Z* = −3.577, *p* < 0.001, *r* = −0.487).

**Figure 4:**
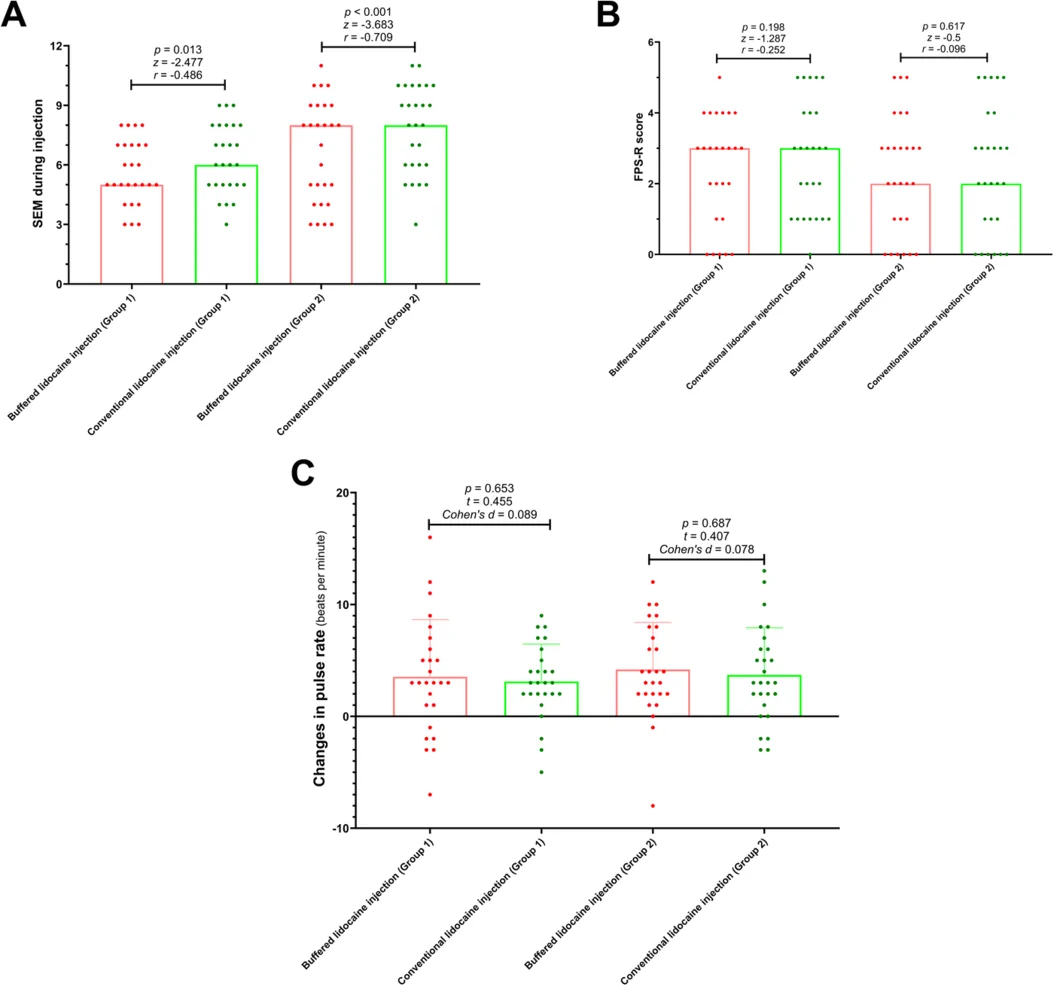
Measuring the pain during the injection of local anesthesia among participants. (A) SEM scores (median). (B) FPS‐R scores (median). (C) Changes in pulse rate (mean [SD]). Each individual is represented as a colored circle. Data in (A) and (B) were analyzed with the Wilcoxon signed ranks test; *r* values indicate effect size. Data in (C) were analyzed with the paired samples *t*‐test; Cohen's *d* values indicate effect size. FPS‐R = faces pain scale‐revised; SEM = sound, eye, and motor.

Additionally, the findings related to pain during injection are detailed in Figure 4.

SEM during injection (Figure 4A). Median SEM scores were significantly lower with buffered lidocaine than with conventional lidocaine in both sequence groups. In Group 1, the median SEM score decreased from 6.0 (IQR 5.0–8.0) after conventional lidocaine to 5.0 (IQR 4.8–7.0) after buffered lidocaine (*Z* = −2.477, *p* = 0.013, *r* = −0.486). In Group 2, the median SEM score after conventional lidocaine was 8.0 (IQR 4.0–9.0), compared with 8.0 (IQR 6.0–10.0) after buffered lidocaine; despite the identical medians, the distribution of scores shifted significantly in favor of buffered lidocaine (*Z* = −3.683, *p* < 0.001, *r* = −0.709).

FPS‐R pain scores (Figure 4B). FPS‑R scores did not differ significantly between the two anesthetic formulations. In Group 1, the median FPS‑R score was 3.0 (IQR 1.0–4.0) for both conventional and buffered lidocaine (*Z* = −1.287, *p* = 0.198, *r* = −0.252). In Group 2, the median FPS‑R score was 2.0 (IQR 1.0–3.0) for conventional and 2.0 (IQR 1.0–4.0) for buffered lidocaine (*Z* = −0.500, *p* = 0.617, *r* = −0.096).

Pulse rate change (Figure 4C). Pulse rate increased after injection across all conditions (mean increases: 3.11–4.18 bpm). However, the magnitude of this increase did not differ between the two anesthetic formulations. Within Group 1, the mean pulse rate change was similar after conventional lidocaine (3.11 (3.33) bpm) and after buffered lidocaine (3.54 (5.11) bpm; paired *t*(25) = 0.455, *p* = 0.653, Cohen's *d* = 0.089). Within Group 2, the mean change after buffered lidocaine (4.18 (4.21) bpm) did not significantly differ from that after conventional lidocaine (3.70 (4.21) bpm; paired *t*(26) = 0.407, *p* = 0.687, Cohen's *d* = 0.078).

### 3.4 Anesthetic Depth and Duration of LA

**Figure 5:**
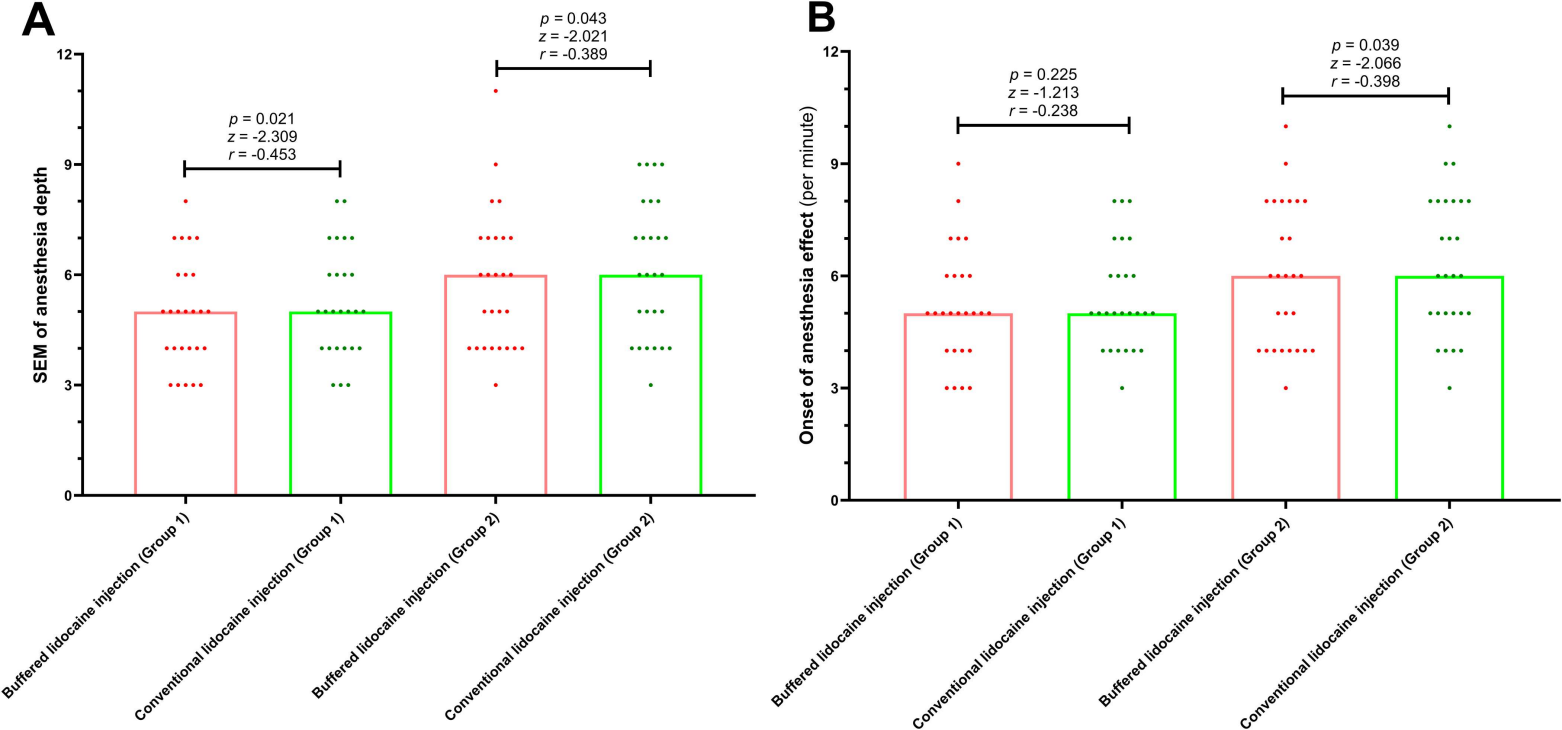
Comparing the depth of local anesthesia (A) and the onset of local anesthesia effect (B) between sessions of each group. Each individual is represented as a colored circle. Data were analyzed with the Wilcoxon signed ranks test; *r* values indicate effect size. SEM = sound, eye, and motor.

The effectiveness of LA with respect to anesthetic depth and duration of LA is shown in Figure 5.

SEM depth (Figure 5A). Median SEM depth scores were significantly lower with buffered lidocaine than with conventional lidocaine in both sequence groups. In Group 1, the median SEM depth was 5.0 (IQR 4.0–6.3) after conventional lidocaine and 5.0 (IQR 4.0–6.0) after buffered lidocaine (Wilcoxon *Z* = −2.309, *p* = 0.021, *r* = −0.453). In Group 2, the median SEM depth was 6.0 (IQR 4.0–7.0) after conventional lidocaine and 6.0 (IQR 4.0–8.0) after buffered lidocaine; despite the identical medians, the distribution shifted significantly in favor of buffered lidocaine (*Z* = −2.021, *p* = 0.043, *r* = −0.389).

Onset of anesthesia (Figure 5B). Onset time was generally shorter with buffered lidocaine. In Group 1, the median onset time was 5.0 min (IQR 4.0–6.3) after conventional lidocaine and 5.0 min (IQR 4.0–6.0) after buffered lidocaine (Wilcoxon *Z* = −1.213, *p* = 0.225, *r* = −0.238). In Group 2, the median onset time decreased significantly from 6.0 min (IQR 4.0–8.0) after conventional lidocaine to 6.0 min (IQR 5.0–8.0) after buffered lidocaine (*Z* = −2.066, *p* = 0.039, *r* = −0.398).

### 3.5 Anesthetic Efficacy Comparison Based on Demographic

Comparison of LA efficacy and physiological responses across different tooth targets (Supporting Information S1: Table S1), dental procedures (Supporting Information S1: Table S2), jaw position (Supporting Information S1: Table S3), and gender (Supporting Information S1: Table S4) is provided in the Supporting Information.

### 3.6 GLMM Analysis of Predictors of Clinical Outcomes

GLMMs were fitted to evaluate the effects of treatment, sequence, session, and clinical covariates on five key outcomes. Full model specifications, goodness‑of‑fit indices, and effect‑size measures are provided in Supporting Information S1: Tables S5 and S6.

*SEM during injection*. The conventional lidocaine injection produced significantly higher SEM scores than the buffered injection, corresponding to a 17% increase (Exp(*β*) = 1.17, 95% CI [1.10, 1.23], *p* < 0.001). A significant sequence effect was also observed: Group 1 (Conventional then Buffered) had 17% lower SEM scores than Group 2 (Buffered then Conventional; Exp(*β*) = 0.83, 95% CI [0.69, 0.99], *p* = 0.042). Session, gender, age, tooth type, jaw position, and dental procedure were not significant predictors.

*SEM depth*. Conventional lidocaine also produced significantly deeper SEM scores (6.6% increase; Exp(*β*) = 1.07, 95% CI [1.03, 1.11], *p* = 0.001). Group 1 again showed significantly lower scores (16% reduction; Exp(*β*) = 0.84, 95% CI [0.71, 1.00], *p* = 0.046). No other covariate reached significance.

*Onset time*. The conventional solution prolonged onset time by 5.9% (Exp(*β*) = 1.06, 95% CI [1.01, 1.11], *p* = 0.011). The sequence effect (group) and gender showed non‑significant trends (*p* = 0.079 and *p* = 0.087, respectively), while session and age were not significant.

*Pulse rate change*. The GLMM indicated no significant effects of treatment, group, session, gender, age, or dental covariates on pulse rate change; the overall model was not significant (*F*(13, 92) = 0.36, *p* = 0.979), and the standardized treatment effect was negligible (Cohen's *d* = –0.10). This confirms that the hemodynamic response did not differ between the two anesthetic formulations.

*FPS‑R pain scores*. Gender and dental procedure type were significant predictors. Female gender was associated with markedly higher cumulative odds of reporting greater pain (Exp(*β*) = 17.75, 95% CI [1.56, 202.42], *p* = 0.021). Relative to the restoration reference, the extraction procedure (Exp(*β*) = 1921.99, 95% CI [1.08, 3425786], *p* = 0.048) and apexogenesis procedure (Exp(*β*) = 816.77, 95% CI [3.50, 190395], *p* = 0.017) were associated with substantially higher odds of a greater pain rating. Treatment, group, session, and age were not significant predictors.

## 4 Discussion

This crossover study assessed the efficacy of buffered lidocaine compared to conventional lidocaine in 53 pediatric dental patients. By applying a GLMM approach, we were able to distinguish the treatment effect from the impact of administration order. Our results show that buffering 2% lidocaine with 7.5% sodium bicarbonate significantly reduces objective signs of distress (SEM score) and shortens LA onset time, compared to lidocaine with epinephrine alone. Conventional treatment was consistently associated with higher SEM scores (~17%) and slower onset times (~6% longer).

A significant sequence effect (group) was observed for SEM during injection and SEM depth, with Group 1 (Conventional then Buffered) showing lower distress scores than Group 2. This indicates that the order of administration influenced the outcomes, but the treatment effect remained significant after adjusting for this sequence effect. For onset time, the sequence effect was a non‑significant trend (*p* = 0.079).

The descriptive analyses displayed in Figures 4 and 5 reinforce these findings. Median SEM scores during injection (Figure 4A) and SEM depth (Figure 5A) were significantly lower with buffered lidocaine in both groups, with effect sizes (*r*) ranging from −0.39 to −0.71. Onset time (Figure 5B) also favored buffered lidocaine, though the difference reached significance only in Group 2 (*p* = 0.039). The GLMM results (Supporting Information S1: Table S5) confirm that these treatment effects were robust after controlling for sequence, session, and clinical covariates.

Gender and dental procedure type were significant predictors in the FPS‑R model (Supporting Information S1: Table S6), but the treatment effect was not. Female gender was associated with markedly higher cumulative odds of reporting greater pain (Exp(*β*) = 17.75, *p* = 0.021). Notably, relative to the restoration reference, the extraction and apexogenesis procedures were associated with dramatically elevated odds of higher pain ratings (Exp(*β*) = 1922 and 817, respectively; *p* < 0.05 for both).

Notably, the subjective FPS‐R scores did not differ between buffered and conventional lidocaine (Figure 4B), whereas the objective SEM scores strongly favored the buffered solution (Figure 4A). This discrepancy may be attributed to the inherent limitations of self‐report measures in pediatric patients, such as varying cognitive maturity and the potential for children to mask pain (Jaaniste et al. 2016; Chan and von Baeyer 2016). The SEM scale, as an objective behavioral observation tool, may therefore provide a more sensitive measure of immediate distress in this age group by capturing involuntary motor and vocal responses (Aminabadi et al. 2008). This divergence between behavioral observation and self‑report is well documented in the pediatric pain literature (Beyer et al. 1990). Our finding of reduced injection pain appears consistent with previous studies using 7.5% sodium bicarbonate, such as Agrawal et al. (2022), who reported significantly lower VAS scores for mandibular blocks. However, the literature shows discrepancies regarding onset and duration: Subburn et al. (2023) observed faster onset and longer duration, while Agrawal et al. found no significant differences. These variations may be due to differences in study design, injection technique, or patient factors. Although limited by sample size, our pediatric split‐mouth trial, which controls for patient variables and includes multiple procedures, may help clarify the clinical profile of 7.5% buffered lidocaine in pediatric dentistry. Recently, a 2025 study reported similar findings in an adult population undergoing dental extractions, with buffered lidocaine (8.4% sodium bicarbonate) producing significantly lower VAS pain scores, faster onset, and longer duration compared to conventional lidocaine. These results, together with our pediatric data, suggest that the clinical advantages of buffered lidocaine extend across age groups (Hamad et al. 2025).

A study by Gorrela et al. found that buffered LA provides a faster onset of anesthesia, a longer duration of effect, and decreased pain during injection (Gorrela et al. 2024). Moreover, a study using the VAS method in an adult group demonstrated that alkalized LA solutions containing sodium bicarbonate are effective at minimizing injection pain and facilitating a more rapid onset of anesthesia (Kashyap et al. 2011). However, some studies did not find a notable association between buffered lidocaine and a reduction in pain during injection (Rabinowitz et al. 2024). For example, a double‐blind crossover study involving children aged 6–12 years who received inferior alveolar nerve blocks found that buffered 2% lidocaine did not result in significant differences in pain scores, as measured by the SEM scale and the Heft‐Parker visual analog scale, compared with unbuffered lidocaine. The onset time was also unchanged in their study (Chopra et al. 2016).

Jain et al. (2022) demonstrated that buffered lidocaine with 8.4% sodium bicarbonate resulted in a quicker onset, prolonged anesthesia, and diminished early postoperative discomfort, whereas both buffered and non‐buffered solutions exhibited comparable intraoperative effectiveness for nerve blocks.

Alongside pharmacologic adjustments like buffering lidocaine to alleviate injection discomfort, non‐pharmacologic approaches could also improve patient comfort during the administration of LA in pediatric cases. A recent randomized crossover study showed that the combination of external cold application and a commercially available vibrating device significantly alleviated both subjective and objective measures of discomfort and fear in children receiving maxillary infiltration injections with conventional lidocaine. Children reported reduced pain levels on the Wong–Baker scale, while technicians noted lower heart rates and FLACC scores compared with conventional injection methods (Alanazi et al. 2019).

Pulse rate was significantly higher after injection than before injection across all conditions (Figure 3). However, based on the GLMM (Table S6) and the paired *t*‑tests (Figure 4C), the magnitude of this increase did not differ between treatments, sessions, or sequence groups. The negligible effect sizes (paired *t*‑test Cohen's *d* = 0.089; GLMM Cohen's *d* = −0.10) reinforce that buffered lidocaine does not alter the cardiovascular response to LA.

Our study has some limitations. The primary focus of the study was on immediate outcomes, such as anesthetic depth, onset, and injection discomfort. Long‐term effects, such as the duration of LA or postoperative pain, were not assessed and may warrant further research. The participants in this study were healthy children receiving treatment at a single academic dental center; consequently, the results may not be entirely applicable to children with systemic conditions, special healthcare needs, or those receiving treatment in private practice or various clinical environments. While a split‐mouth, crossover design minimizes inter‐individual variability, supplementary analyses comparing anesthetic efficacy across tooth targets (Supporting Information S1: Table S1), dental procedures (Supporting Information S1: Table S2), jaw position (Supporting Information S1: Table S3), and gender (Supporting Information S1: Table S4) revealed no consistent significant differences in SEM scores, onset time, pulse rate change, or FPS‑R ratings across these subgroups. The few isolated significant results were small in magnitude and did not alter the primary finding that buffered lidocaine outperformed conventional lidocaine, confirming that the treatment effect was not confounded by these baseline characteristics. Additionally, despite our efforts to implement a double‑blind protocol, the potential for residual bias in subjective pain assessment remains a limitation, as complete blinding of pediatric participants is difficult to achieve. Furthermore, the trial was registered retrospectively, which deviates from the standard of prospective registration. To minimize the risk of selective outcome reporting, the study was conducted strictly according to a protocol prospectively approved by the Kerman Research Ethics Committee on June 6, 2022, in which all outcomes and analyses were pre‑specified and remained unchanged. However, because the public record was created after the results were known, some caution in interpretation is warranted. To handle potential carryover and period effects, session and group were entered as fixed covariates in the GLMM. An additional variable ‘Sequence’ was included but was non‑estimable (df = 0) due to aliasing with group. Session was not significant in any model, indicating no period effect; group was significant for two objective outcomes, confirming a sequence effect that was fully adjusted for in the estimation of treatment effects. Finally, the generalizability of our findings is limited by the study population and setting. As the participants were healthy children treated within a single academic dental center, the results may not be directly applicable to children with special healthcare needs, systemic medical conditions, or those receiving treatment in diverse clinical environments, such as private practices.

## 5 Conclusion

In this randomized, split‑mouth, crossover trial involving healthy children, buffering 2% lidocaine with 7.5% sodium bicarbonate significantly reduced objective behavioral distress—reflected in lower SEM scores and faster anesthetic onset—compared with conventional lidocaine. The subjective pain experience reported by children (FPS‑R) did not differ between the two local anesthetic formulations, underscoring that objective behavioral indicators and self‑reported pain capture distinct dimensions of the injection experience in this age group. The reduction in physical distress behaviors supports the use of buffered lidocaine as a practical strategy to improve the injection experience in pediatric dentistry. These findings are derived from a single academic center and a healthy pediatric population; further research in broader populations and with long‑term outcomes is warranted.

## Author Contributions

Project administration: Raziyeh Shojaeipour. Resources: Raziyeh Shojaeipour, Sajjad Amiri, and Abolfazl Shiri Varnamkhasti. Funding acquisition: Raziyeh Shojaeipour and Sajjad Amiri. Supervisions: Raziyeh Shojaeipour and Milad Mollaali. Conceptualization: Raziyeh Shojaeipour, Sajjad Amiri, Abolfazl Shiri Varnamkhasti, and Milad Mollaali. Writing manuscript draft: Milad Mollaali. Investigations and sample collection: Sajjad Amiri, Abolfazl Shiri Varnamkhasti, and Raziyeh Shojaeipour. Methodology: Raziyeh Shojaeipour, Milad Mollaali, Sajjad Amiri, and Abolfazl Shiri Varnamkhasti. Formal analysis: Milad Mollaali and Sajjad Amiri. Software: Milad Mollaali. Data curation: Milad Mollaali and Raziyeh Shojaeipour. Visualization: Milad Mollaali. Critical reviewing and editing of the final manuscript: Raziyeh Shojaeipour, Milad Mollaali, Abolfazl Shiri Varnamkhasti, and Sajjad Amiri. Approving manuscript contents: Abolfazl Shiri Varnamkhasti, Sajjad Amiri, Raziyeh Shojaeipour, and Milad Mollaali.

## Ethics Statement

Ethics approval was obtained from the Ethics Committee of Kerman University of Medical Sciences under the code: IR.KMU.REC.1401.161.

## Consent

Informed written consent was obtained from the parents of all child participants before their enrollment in the research.

## Conflicts of Interest

The authors declare no conflicts of interest.

## Supporting information


Supporting File

## Data Availability

The data that support the findings of this study are available from the corresponding author upon reasonable request.
