# Supplementary material for: Effectiveness of Buffered Lidocaine With Sodium Bicarbonate Versus Conventional Lidocaine as Local Anesthetic Solutions for Pain Management in Pediatric Dentistry: A Randomized, Split‐Mouth, Crossover, Double‐Blind Clinical Trial
Source: Clin Exp Dent Res. 2026 Aug 25;12(4):e70435. doi: 10.1002/cre2.70435 (PMC13506132; doi:10.1002/cre2.70435)
Supplement: Supplementary file 1 [file CRE2-12-e70435-s001.docx]

Supplementary material

**Effectiveness of Buffered Lidocaine with Sodium Bicarbonate Versus Conventional Lidocaine as Local Anesthetic Solutions for Pain Management in Pediatric Dentistry: A Randomized, Split-Mouth, Crossover, Double-Blind Clinical Trial**

Running title: Buffered Lidocaine for Pain Management in Children

**Table S1.** Comparative analysis of anesthetic efficacy and physiological responses across the tooth targets in the study groups.

|  | **Group 1** (*N*=26) | | | |  | **Group 2** (*N*=27) | | | |
| --- | --- | --- | --- | --- | --- | --- | --- | --- | --- |
| **Variables** | ***Median*** | ***H*** | ***p*** ^a^ | ***η^2^*** ^d,†^ |  | ***Median*** | ***H*** | ***p*** | ***η^2^*** |
| SEM during injection at the first session | 6 | 3.265 | 0.195 | 0.055 |  | 8 | 5.96 | 0.051 | 0.165 |
| SEM during injection at the second session | **5** | **6.246** | **0.044^*^** | **0.192** |  | 8 | 3.583 | 0.167 | 0.066 |
| SEM of anesthesia’s depth at the first session | 5 | 2.563 | 0.278 | 0.024 |  | 6 | 4.323 | 0.115 | 0.097 |
| SEM of anesthesia’s depth at the second session | 5 | 0.710 | 0.701 | 0.000 |  | 6 | 3.1 | 0.212 | 0.046 |
| Onset of anesthesia’s effect at the first session | 5 | 1.090 | 0.580 | 0.000 |  | **6** | **7.207** | **0.027^*^** | **0.217** |
| Onset of anesthesia’s effect at the second session | 5 | 2.765 | 0.251 | 0.033 |  | 6 | 5.661 | 0.059 | 0.152 |
| **Variables** | **Total *Mean* (*SD*)** | ***F*** | ***p*** ^b^ | ***η^2^*** ^e^ |  | **Total *Mean* (*SD*)** | ***F*** | ***p*** | ***η^2^*** |
| Changes in pulse rate at the first session | 3.115 (3.338) | 1.233 | 0.310 | 0.097 |  | 4.185 (4.206) | 0.259 | 0.774 | 0.021 |
| Changes in pulse rate at the second session | 3.538 (5.109) | 0.252 | 0.779 | 0.021 |  | 3.704 (4.214) | 1.037 | 0.370 | 0.08 |
| **Variables** | ***df*** | ***χ^2^*** | ***p*** ^c^ | **Cramer’s *V*** ^f^ |  | ***df*** | ***χ^2^*** | ***p*** | **Cramer’s *V*** |
| FPS-R at the first session | 10 | 6.9 | 0.735 | 0.364 |  | 10 | 3.512 | 0.967 | 0.255 |
| FPS-R at the Second session | 10 | 10.075 | 0.434 | 0.44 |  | 10 | 15.105 | 0.128 | 0.529 |

*Note.* SEM = Sound, Eye, and Motor; FPS-R = Faces Pain Scale-Revised; a = Kruskal-Wallis Test; b = One-Way ANOVA Test; c = Chi-Square Test of Independence; d, e, f = Effect size; † = The formula (*η²* = (*H* − k + 1) / (n − k)) can yield negative estimates when the effect is negligible, which are reported as 0.000; * *p* < 0.05.

**Table S2.** Comparative analysis of anesthetic efficacy and physiological responses across dental procedures in study groups.

|  | **Group 1** (*N*=26) | | | |  | **Group 2** (*N*=27) | | | |
| --- | --- | --- | --- | --- | --- | --- | --- | --- | --- |
| **Variables** | ***Median*** | ***H*** | ***p*** ^a^ | ***η^2^*** ^d, †^ |  | ***Median*** | ***H*** | ***p*** | ***η^2^*** |
| SEM during injection at the first session | 6 | 6.899 | 0.228 | 0.095 |  | **8** | **9.816** | **0.044^*^** | **0.229** |
| SEM during injection at the second session | 5 | 7.020 | 0.219 | 0.101 |  | **8** | **9.632** | **0.047^*^** | **0.220** |
| SEM of anesthesia’s depth at the first session | 5 | 5.217 | 0.390 | 0.011 |  | 6 | 5.446 | 0.245 | 0.021 |
| SEM of anesthesia’s depth at the second session | 5 | 4.016 | 0.547 | 0.000 |  | 6 | 4.933 | 0.294 | 0.000 |
| Onset of anesthesia’s effect at the first session | 5 | 5.641 | 0.343 | 0.032 |  | 6 | 7.369 | 0.118 | 0.113 |
| Onset of anesthesia’s effect at the second session | 5 | 4.335 | 0.502 | 0.000 |  | 6 | 9.952 | 0.041^*^ | 0.236 |
| **Variables** | **Total *Mean* (*SD*)** | ***F*** | ***p*** ^b^ | ***η^2^*** ^e^ |  | **Total *Mean* (*SD*)** | ***F*** | ***p*** | ***η^2^*** |
| Changes in pulse rate at the first session | 3.115 (3.338) | 1.097 | 0.393 | 0.215 |  | 4.185 (4.206) | 0.346 | 0.844 | 0.059 |
| Changes in pulse rate at the second session | 3.538 (5.109) | 0.093 | 0.992 | 0.023 |  | 3.704 (4.214) | 0.688 | 0.608 | 0.111 |
| **Variables** | ***df*** | ***χ^2^*** | ***p*** ^c^ | **Cramer’s *V*** ^f^ |  | ***df*** | ***χ^2^*** | ***p*** | **Cramer’s *V*** |
| FPS-R at the first session | 25 | 28.329 | 0.293 | 0.467 |  | 20 | 15.920 | 0.722 | 0.384 |
| FPS-R at the second session | 25 | 28.078 | 0.304 | 0.465 |  | 20 | 16.152 | 0.707 | 0.387 |

*Note.* SEM = Sound, Eye, and Motor; FPS-R = Faces Pain Scale-Revised; a = Kruskal-Wallis Test; b = One-Way ANOVA Test; c = Chi-Square Test of Independence; d, e, f = Effect size; † = The formula (*η²* = (*H* − k + 1) / (n − k)) can yield negative estimates when the effect is negligible, which are reported as 0.000; * *p* < 0.05.

**Table S3.** Comparative analysis of anesthetic efficacy and physiological responses across jaw positions in study groups.

|  | **Group 1** (*N*=26) | | | | |  | **Group 2** (*N*=27) | | | | |
| --- | --- | --- | --- | --- | --- | --- | --- | --- | --- | --- | --- |
| **Variables** | ***Median*** | ***U*** | ***z*** | ***p*** ^a^ | ***r*** ^d^ |  | ***Median*** | ***U*** | ***z*** | ***p*** | ***r*** |
| SEM during injection at the first session | 6 | 72 | -0.428 | 0.669 | -0.084 |  | 8 | 59 | -1.570 | 0.116 | -0.302 |
| SEM during injection at the second session | 5 | 72 | -0.431 | 0.666 | -0.084 |  | 8 | 60.5 | -1.499 | 0.134 | -0.288 |
| SEM of anesthesia’s depth at the first session | 5 | 55.5 | -1.318 | 0.188 | -0.258 |  | 6 | 57.5 | -1.660 | 0.097 | -0.319 |
| SEM of anesthesia’s depth at the second session | 5 | 57.5 | -1.212 | 0.225 | -0.238 |  | **6** | **43.5** | **-2.337** | **0.019^*^** | **-0.45** |
| Onset of anesthesia’s effect at the first session | 5 | 67.5 | -0.679 | 0.497 | -0.133 |  | 6 | 52 | -1.936 | 0.053 | -0.372 |
| Onset of anesthesia’s effect at the second session | 5 | 59.5 | -1.110 | 0.267 | -0.218 |  | **6** | **45.5** | **-2.241** | **0.025^*^** | **-0.431** |
| **Variables** | **Total *Mean* (*SD*)** | | ***t*** | ***p*** ^b^ | **Cohen’s *d*** ^e^ |  | **Total *Mean* (*SD*)** | | ***t*** | ***p*** | **Cohen’s *d*** |
| Changes in pulse rate at the first session | 3.115 (3.338) | | 0.137 | 0.893 | 0.055 |  | 4.185 (4.206) | | 1.143 | 0.264 | 0.44 |
| Changes in pulse rate at the second session | 3.538 (5.109) | | -1.246 | 0.225 | -0.502 |  | 3.704 (4.214) | | 0.013 | 0.990 | 0.005 |
| **Variables** | ***df*** | | ***χ^2^*** | ***p*** ^c^ | **Cramer’s *V*** ^f^ |  | ***df*** | | ***χ^2^*** | ***p*** | **Cramer’s *V*** |
| FPS-R at the first session | 5 | | 4.634 | 0.462 | 0.422 |  | 5 | | 10.062 | 0.073 | 0.61 |
| FPS-R at the second session | 5 | | 4.840 | 0.436 | 0.431 |  | 5 | | 5.637 | 0.343 | 0.457 |

*Note.* SEM = Sound, Eye, and Motor; FPS-R = Faces Pain Scale-Revised; a = Mann-Whitney *U* Test; b = Independent Samples *t*-Test; c = Chi-Square Test of Independence; d, e, f = Effect size; * *p* < 0.05.

**Table S4.** Comparative analysis of anesthetic efficacy and physiological responses across gender in study groups.

|  | **Group 1** (*N*=26) | | | | |  | **Group 2 (***N*=27) | | | | | |
| --- | --- | --- | --- | --- | --- | --- | --- | --- | --- | --- | --- | --- |
| **Variables** | ***Median*** | ***U*** | ***z*** | ***p*** ^a^ | ***r*** ^d^ |  | ***Median*** | ***U*** | | ***z*** | ***p*** | ***r*** |
| SEM during injection at the first session | 6 | 68.5 | -0.737 | 0.461 | -0.144 |  | 8 | 84 | | -0.344 | 0.731 | -0.066 |
| SEM during injection at the second session | 5 | 69.5 | -0.69 | 0.490 | -0.135 |  | 8 | 86.5 | | -0.221 | 0.825 | -0.042 |
| SEM of anesthesia’s depth at the first session | 5 | 73 | -0.503 | 0.615 | -0.099 |  | 6 | 78 | | -0.644 | 0.519 | -0.124 |
| SEM of anesthesia’s depth at the second session | 5 | 77 | -0.292 | 0.770 | -0.057 |  | 6 | 65.5 | | -1.255 | 0.210 | -0.241 |
| Onset of anesthesia’s effect at the first session | 5 | 80 | -0.134 | 0.894 | -0.026 |  | 6 | 67.5 | | -1.166 | 0.243 | -0.224 |
| Onset of anesthesia’s effect at the second session | 5 | 67.5 | -0.8 | 0.424 | -0.157 |  | 6 | 58 | | -1.625 | 0.104 | -0.313 |
| **Variables** | **Total *Mean* (*SD*)** | | ***t*** | ***p*** ^b^ | **Cohen’s *d*** ^e^ |  | **Total *Mean* (*SD*)** | | ***t*** | | ***p*** | **Cohen’s *d*** |
| Changes in pulse rate at the first session | 3.115 (3.338) | | 0.916 | 0.369 | 0.364 |  | 4.185 (4.206) | | 0.053 | | 0.958 | 0.02 |
| Changes in pulse rate at the second session | 3.538 (5.109) | | -0.07 | 0.945 | -0.028 |  | 3.704 (4.214) | | 0.437 | | 0.666 | 0.168 |
| **Variables** | ***df*** | | ***χ^2^*** | ***p*** ^c^ | **Cramer’s *V*** ^f^ |  | ***df*** | | ***χ^2^*** | | ***p*** | **Cramer’s *V*** |
| FPS-R at the first session | 5 | | 5.457 | 0.363 | 0.458 |  | 5 | | 4.646 | | 0.461 | 0.415 |
| FPS-R at the second session | 5 | | 10.090 | 0.073 | 0.623 |  | 5 | | 5.637 | | 0.343 | 0.457 |

*Note.* SEM = Sound, Eye, and Motor; FPS-R = Faces Pain Scale-Revised; a = Mann-Whitney *U* Test; b = Independent Samples *t*-Test; c = Chi-Square Test of Independence; d, e, f = Effect size.

**Table S5.** Generalized Linear Mixed Model (GLMM) Estimates for Objective Clinical Outcomes.

| **Predictor** | **SEM during injection**  (Gamma Distribution, Log Link) | | | |  | **SEM depth**  (Gamma Distribution, Log Link) | | | |  | **Onset time**  (Gamma Distribution, Log Link) | | | |
| --- | --- | --- | --- | --- | --- | --- | --- | --- | --- | --- | --- | --- | --- | --- |
|  | ***β* (*SE*)** | ***t*** | ***p*** | **Exp*(β)* [95% CI]** |  | ***β* (*SE*)** | ***t*** | ***p*** | **Exp*(β)* [95% CI]** |  | ***β* (*SE*)** | ***t*** | ***p*** | **Exp*(β)* [95% CI]** |
| **Fixed Effects** |  | | | |  |  | | | |  |  | | | |
| Intercept | 1.57 (0.29) | 5.39 | <0.001 | 4.81 [2.69, 8.60] |  | **1.48 (0.28)** | **5.26** | **<0.001^***^** | **4.40 [2.52, 7.71]** |  | **1.49 (0.26)** | **5.82** | **<0.001^***^** | **4.45 [2.67, 7.41]** |
| Conventional (vs. Buffered) | **0.15 (0.03)** | **5.31** | **<0.001^***^** | **1.17 [1.10, 1.23]** |  | **0.06 (0.02)** | **3.52** | **0.001^**^** | **1.07 [1.03, 1.11]** |  | **0.06 (0.02)** | **2.58** | **0.011^*^** | **1.06 [1.01, 1.11]** |
| Group 1 (vs. Group 2) | **-0.19 (0.09)** | **-2.07** | **0.042^*^** | **0.83 [0.69, 0.99]** |  | **-0.18 (0.09)** | **-2.02** | **0.046^*^** | **0.84 [0.71, 1.00]** |  | -0.14 (0.08) | -1.78 | 0.079 | 0.87 [0.74, 1.02] |
| First session (vs. Second) | -0.03 (0.03) | -1.16 | 0.250 | 0.97 [0.91, 1.02] |  | 0.00 (0.02) | 0.02 | 0.986 | 1.00 [0.97, 1.04] |  | -0.00 (0.02) | -0.12 | 0.907 | 1.00 [0.95, 1.04] |
| Female (vs. Male) | 0.01 (0.09) | 0.12 | 0.902 | 1.01 [0.84, 1.21] |  | 0.09 (0.09) | 1.05 | 0.297 | 1.10 [0.92, 1.31] |  | 0.14 (0.08) | 1.73 | 0.087 | 1.15 [0.98, 1.35] |
| Age | 0.01 (0.03) | 0.27 | 0.785 | 1.01 [0.95, 1.07] |  | 0.00 (0.03) | 0.08 | 0.935 | 1.00 [0.94, 1.06] |  | -0.00 (0.03) | -0.07 | 0.942 | 1.00 [0.95, 1.05] |
| Tooth D (vs. Tooth 6) | -0.02 (0.26) | -0.09 | 0.930 | 0.98 [0.58, 1.64] |  | 0.23 (0.25) | 0.93 | 0.357 | 1.26 [0.77, 2.08] |  | 0.12 (0.23) | 0.50 | 0.618 | 1.12 [0.71, 1.77] |
| Tooth E (vs. Tooth 6) | -0.11 (0.26) | -0.41 | 0.683 | 0.90 [0.54, 1.51] |  | 0.04 (0.25) | 0.18 | 0.860 | 1.04 [0.63, 1.73] |  | -0.04 (0.23) | -0.15 | 0.879 | 0.97 [0.61, 1.52] |
| Maxilla (vs. Mandible) | 0.01 (0.10) | 0.14 | 0.891 | 1.01 [0.83, 1.24] |  | 0.08 (0.10) | 0.80 | 0.424 | 1.08 [0.89, 1.31] |  | 0.07 (0.09) | 0.75 | 0.456 | 1.07 [0.90, 1.27] |
| Extraction (vs. Restoration) | 0.43 (0.25) | 1.71 | 0.091 | 1.54 [0.93, 2.56] |  | 0.01 (0.25) | 0.03 | 0.978 | 1.01 [0.62, 1.64] |  | 0.22 (0.22) | 0.99 | 0.327 | 1.25 [0.80, 1.95] |
| Crown (vs. Restoration) | 0.32 (0.41) | 0.78 | 0.439 | 1.37 [0.61, 3.06] |  | -0.16 (0.39) | -0.41 | 0.683 | 0.85 [0.39, 1.86] |  | 0.15 (0.36) | 0.43 | 0.670 | 1.17 [0.57, 2.36] |
| Pulpotomy (vs. Restoration) | 0.39 (0.24) | 1.60 | 0.113 | 1.47 [0.91, 2.37] |  | 0.00 (0.23) | 0.02 | 0.988 | 1.00 [0.63, 1.59] |  | 0.13 (0.21) | 0.60 | 0.547 | 1.14 [0.75, 1.73] |
| Pulpectomy (vs. Restoration) | 0.40 (0.27) | 1.48 | 0.143 | 1.49 [0.87, 2.53] |  | 0.21 (0.26) | 0.80 | 0.428 | 1.23 [0.73, 2.06] |  | 0.31 (0.24) | 1.31 | 0.195 | 1.36 [0.85, 2.17] |
| Apexogenesis (vs. Restoration) | 0.05 (0.21) | 0.22 | 0.825 | 1.05 [0.69, 1.58] |  | 0.06 (0.20) | 0.32 | 0.752 | 1.06 [0.72, 1.58] |  | 0.09 (0.18) | 0.48 | 0.629 | 1.09 [0.76, 1.57] |
| **Random effects** |  | | | |  |  | | | |  |  | | | |
| Subject (Intercept) | **Var** = 0.085, ***Z*** = 3.99, ***p*** < 0.001^***^ | | | |  | **Var** = 0.086, ***Z*** = 4.31, ***p*** < 0.001^***^ | | | |  | **Var** = 0.068, ***Z*** = 4.12, ***p*** < 0.001^***^ | | | |
| Residual (φ) | **Var** = 0.022, ***Z*** = 5.05, ***p*** < 0.001^***^ | | | |  | **Var** = 0.009, ***Z*** = 5.05, ***p*** < 0.001^***^ | | | |  | **Var** = 0.013, ***Z*** = 5.05, ***p*** < 0.001^***^ | | | |
| **Model Fit** | **AICc** = 43.275, **BIC** = 48.184, ***R^2^*_pred_** = 0.906 | | | |  | **AICc** = -7.137, **BIC** = -2.228, ***R^2^*_pred_** = 0.951 | | | |  | **AICc** = 6.157, **BIC** = 11.065, ***R^2^*_pred_** = 0.933 | | | |

*Note.* *N* = 106. SEM = Sound, Eyes, Motor. φ = Gamma scale parameter. *R^2^*_pred_ = squared Pearson correlation between observed and model‑predicted values. Akaike Information Criterion (AICc). Bayesian Information Criterion (BIC). Sequence was non‑estimable (redundant) in all models. All confidence intervals (*CI*) were obtained via the Wald method. * *p* < 0.05.  ** *p* < 0.01.  *** *p* < 0.001.

**Table S6.** Generalized Linear Mixed Model (GLMM) Estimates for Physiological and Subjective Outcomes.

| **Predictor** | **Pulse rate change** (Normal, Identity) | | | |  | **FPS-R score (**Multinomial, Cumulative Logit) | | | |
| --- | --- | --- | --- | --- | --- | --- | --- | --- | --- |
|  | ***β* (*SE*)** | ***t*** | ***p*** | ***95% CI* for *β*** |  | ***β* (*SE*)** | ***t*** | ***p*** | **Exp(*β*) [95% *CI*]** |
| **Fixed Effects** |  | | | |  |  | | | |
| Intercept | 5.47 (3.30) | 1.66 | 0.101 | [-1.08, 12.03] |  | — | — | — | — |
| Conventional (vs. Buffered) | -0.45 (0.76) | -0.6 | 0.551 | [-1.95, 1.05] |  | 0.55 (0.40) | 1.37 | 0.173 | 1.72 [0.79, 3.79] |
| Group 1 (vs. Group 2) | -0.30 (1.01) | -0.30 | 0.767 | [-2.30, 1.70] |  | 1.76 (1.18) | 1.49 | 0.139 | 5.78 [0.56, 59.77] |
| First session (vs. Second) | 0.03 (0.76) | 0.04 | 0.969 | [-1.47, 1.53] |  | 0.29 (0.40) | 0.74 | 0.458 | 1.34 [0.61, 2.94] |
| Female (vs. Male) | 0.81 (1.03) | 0.79 | 0.434 | [-1.24, 2.86] |  | **2.88 (1.23)** | **2.35** | **0.021^*^** | **17.75 [1.56, 202.42]** |
| Age | -0.25 (0.34) | -0.73 | 0.470 | [-0.93, 0.43] |  | -0.36 (0.40) | -0.89 | 0.376 | 0.70 [0.32, 1.55] |
| Tooth D (vs. Tooth 6) | -3.46 (2.92) | -1.18 | 0.239 | [-9.25, 2.34] |  | -4.03 (3.73) | -1.08 | 0.283 | 0.02 [0.00, 29.45] |
| Tooth E (vs. Tooth 6) | -3.33 (2.92) | -1.14 | 0.257 | [-9.13, 2.47] |  | -5.53 (3.85) | -1.44 | 0.155 | 0.004 [0.00, 8.39] |
| Maxilla (vs. Mandible) | -0.20 (1.11) | -0.18 | 0.855 | [-2.42, 2.01] |  | -0.23 (1.29) | -0.18 | 0.860 | 0.80 [0.06, 10.33] |
| Extraction (vs. Restoration) | 4.32 (2.85) | 1.52 | 0.133 | [-1.34, 9.98] |  | **7.56 (3.77)** | **2.01** | **0.048*** | **1921.99 [1.08, 3425786]** |
| Crown (vs. Restoration) | 2.95 (4.54) | 0.65 | 0.518 | [-6.07, 11.97] |  | 0.73 (5.44) | 0.14 | 0.893 | 2.08 [0.00, 102944] |
| Pulpotomy (vs. Restoration) | 3.56 (2.7) | 1.32 | 0.19 | [-1.80, 8.92] |  | 4.14 (3.54) | 1.17 | 0.245 | 62.78 [0.06, 70886] |
| Pulpectomy (vs. Restoration) | 2.44 (3) | 0.81 | 0.418 | [-3.52, 8.41] |  | 2.15 (3.87) | 0.56 | 0.579 | 8.62 [0.00, 18793] |
| Apexogenesis (vs. Restoration) | 1.40 (2.32) | 0.61 | 0.546 | [–3.20, 6.00] |  | **6.71 (2.74)** | **2.44** | **0.017*** | **816.77 [3.50, 190395]** |
| **Random Effects** |  | | | |  |  | | | |
| Subject (Intercept) | **Var** = 4.475, ***Z*** = 1.47, ***p*** = 0.143 | | | |  | **Var** = 13.747, ***Z*** = 2.94, ***p*** = 0.003^**^ | | | |
| Residual (σ²) | **Var** = 15.128, ***Z*** = 5.05, ***p*** < 0.001^***^ | | | |  | — | | | |
| **Model Fit** | **AICc** = 574.39, **BIC** = 579.30 | | | |  | **AICc** = 2793.59, **BIC** = 2796.02 | | | |
| **Explained Variance** | **Marginal *R²*** = 0.107, **Conditional *R²*** = 0.311,  **Cohen’s *d* (Conventional vs. Buffered)** = -0.10 | | | |  | **Classification accuracy** = 72.6% | | | |

*Note.* *N* = 106. FPS‑R = Faces Pain Scale‑Revised. For the pulse rate model: Marginal *R²* = variance explained by fixed effects, Conditional *R²* = variance explained by fixed + random effects, Cohen’s *d* = standardized mean difference (Conventional vs. Buffered), and Residual = residual variance (σ²). For the FPS‑R model, the reference category for the ordinal outcome is the highest pain score (5, “Hurts worst”); classification accuracy is the overall percent correct. Akaike Information Criterion (AICc). Bayesian Information Criterion (BIC). Sequence was non‑estimable (redundant) in both models. All confidence intervals (*CI*) were obtained via the Wald method. * *p* < 0.05.  ** *p* < 0.01.  *** *p* < 0.001.
